# Nonautoimmune overt hypothyroidism in the early stages of nephrotic syndrome: a case report from Somalia

**DOI:** 10.1097/MS9.0000000000000450

**Published:** 2023-04-07

**Authors:** Abdisamad Mohamed Adan, Mohamed Osman Siyad, Mohamed Osman Omar Jeele

**Affiliations:** Department of Internal Medicine, Mogadishu Somali Turkish Training and Research Hospital, Mogadishu, Somalia

**Keywords:** nephrotic syndrome, nonautoimmune, overt hypothyroidism, thyroid dysfunction

## Abstract

Nephrotic syndrome (NS) was first characterized in 1827 as the occurrence of proteinuria greater than or equal to 3.5 g/24 h, hypoalbuminemia (albumin≤3.0 g/dl), peripheral edema, hyperlipidemia, lipiduria caused by increased permeability of the renal glomerulus. Persistent proteinuria will eventually lead to hypothyroidism.

**Case presentation:**

In the presenting case, we reported a 26-year-old male patient with no known history of chronic disease who presented to the emergency department with a complaint of 1-week generalized edema, nausea, fatigue, and generalized ache in the extremities. He was diagnosed with NS complicated by hypothyroidism and was hospitalized for 3 weeks. After 3 weeks of treatment and close monitoring, the patient’s clinical condition and laboratory investigations were improved, and was discharged in good health.

**Conclusion:**

Hypothyroidism in the early stages of NS is a rare entity which may be encountered and physicians should be aware that hypothyroidism can be seen at any stage of NS.

## Introduction

Highlights

- Hypothyroidism can present at an early stage of nephrotic syndrome (NS).
- All patients with NS regardless of the stage should be investigated for thyroid dysfunction.
- Increased requirement of levothyroxine dose should not be focused on gastroenterology problems but also include the workup for NS.

NS was first characterized in 1827 as the occurrence of proteinuria greater than or equal to 3.5 g/24 h, hypoalbuminemia (albumin≤3.0 g/dl), peripheral edema, hyperlipidemia, lipiduria caused by increased permeability of the renal glomerulus^1^,^2^. The estimated population incidence of NS is roughly 3 per 100 000 people per year^3^. The increased permeability of the glomerulus can be due to primary causes which include focal segmental glomerulosclerosis, membranous nephropathy, and minimal change disease, and secondary causes which comprise of diabetes, systemic lupus erythematosus, multiple myeloma, amyloidosis, and infections^1^. Although, albumin is the major protein that will be lost in the urine in the case of NS, some other binding proteins like thyroxine-binding globulin and transthyretin, and thyroid hormones itself will be lost resulting in hypothyroidism^4^. In the early stage of NS the loss of thyroid hormones rarely leads to hypothyroidism and a euthyroid state is expected. Aside from the effect of steroid treatment, sick euthyroid on thyroid hormones, persistent proteinuria will eventually lead to hypothyroidism^5^. Nonautoimmune hypothyroidism due to NS which presents at an early stage of NS is quite rare and cases reporting it are very scarce in the literature. Here, we report a case of a 26-year-old male presented with both NS and hypothyroidism at an early stage. Our work has been reported in line with the SCARE 2020 criteria^6^.

## Case report

A 26-year-old, previously healthy man with no known history of chronic disease presented to the emergency department with a complaint of 1-week generalized edema, nausea, fatigue, and generalized ache in the extremities. The patient has no family history of autoimmune diseases. On examination, he looked ill and distressful, and he had a puffy face. His vital signs showed a pulse of 88 bpm, blood pressure of 110/80, a temperature of 36.7°C, and a respiratory rate of 18. Head and neck examinations were normal. Neurological examinations including motor, sensory, and cranial nerves were unremarkable. The cardiovascular examination was also normal. The abdomen was soft with no tenderness and no evidence of organomegaly. He had bilateral grade 3 pitting lower limb edema up to the thighs. He also had sacral and face edema. Laboratory investigations revealed a hemoglobin level of 14 g/dl, glucose 99 mg/dl, aspartate aminotransferase 43 U/l, alanine aminotransferase 35 U/l, urea of 56 mg/dl, creatinine of 0.7 mg/dl, serum sodium 134 mmol/l, and serum potassium of 3.98 mmol/l, total protein 4.7 g/dl, and albumin level of 2.1 g/dl. Regarding the lipid profile, triglyceride was 380 mg/dl, low-density lipoprotein was 290 mg/dl, and total cholesterol was measured at 400 mg/dl. Protein was positive in urinalysis, and the urine protein-to-creatinine ratio resulted 7.27 g of protein per day (Table 1).

**TABLE 1:** Laboratory results from presentation to 2 months of follow-up

Laboratory results	Normal range	On admission	Discharge day (3 weeks later)	1 month after discharge
Hemoglobin (g/dl)	13–17	14	14.6	14
Glucose (mg/dl)	60/110	99	112	100
Urea (mg/dl)	10–45	56	35	22
Creatinine (mg/dl)	0.6–1.35	0.7	0.66	0.63
Aspartate aminotransferase (U/l)	0–35	43	14	20
Alanine aminotransferase (U/l)	0–45	35	28	25
Sodium (mEq/l)	135–145	134	139	141
Potassium (mEq/l)	3.5–5.5	3.98	4.87	4.56
Total cholesterol (mg/dl)	40–200	400	290	180
LDL (mg/dl)	40–130	290	180	112
HDL (mg/dl)	30–70	35	46	60
Triglyceride (mg/dl)	50–200	380	230	198
Total protein (g/dl)	6.4–8.3	4.7	7	7.5
Albumin (g/dl)	3.5–5.5	2.1	3.9	4.2
Thyroid-stimulating hormone (mIU/l)	0.6–5.5	37	9	3.56
Free T4 (ng/ml)	0.7–2.1	0.4	0.78	1.3
Free T3 (pg/ml)	2.4–4.2	2	2.5	2.76
Spot urine protein:creatinine ratio (g/day)	0–3.5	7.27	3.9	1.5

HDL, high-density lipoprotein; LDL, low-density lipoprotein.

A chest radiograph showed mild pleural effusion on both sides of the lungs. Echocardiography and electrocardiogram were both demonstrated normal results. Abdominal ultrasound revealed bilateral normal kidney anatomy with normal parenchyma and mild ascites.

The patient was immediately admitted to the internal medicine unit under the diagnosis of newly onset NS and was started on ramipril 5 mg, methylprednisolone 60 mg/day, furosemide 20 mg, aspirin 100 mg, and atorvastatin 40 mg. On routine blood tests the day after hospitalization, we incidentally revealed hypothyroidism with thyroid-stimulating hormone (TSH) measuring 37 mIU/l and T4 0.4 ng/dl. We tested the thyroid function tests by a Roche e411 Immunoassay Analyzer (Roche Diagnostics Corporation). The antithyroid peroxidase and anti-thyroglobulin antibody tests were negative. The thyroid ultrasound examination was ordered and came back normal. A diagnosis of nonautoimmune overt hypothyroidism due to NS was considered, and levothyroxine treatment was started immediately. A dose of levothyroxine 50 mcg was initially started to lessen side effects and poor compliance and after 3 days it was increased to 100 mcg. After 3 weeks of hospitalization, the patient improved very well and his symptoms subsided. Lower limb edema was decreased drastically, pleural effusion resolved, proteinuria was decreased to 3.9 g/day, and his lipid profile and albumin levels were almost at normal levels. TSH level was also decreased to 9 mIU/l. He was discharged on oral prednisolone, atorvastatin, aspirin, and levothyroxine. After 1 month, the patient came to our clinic for routine follow-up. His TSH level was 3.56 mIU/l, there was no protein in the urine and his albumin and lipid profiles were returned to normal levels.

## Discussion

Second, only to diabetes mellitus in terms of endocrine illnesses, particularly in Africa, thyroid disease is the most prevalent endocrine disorder worldwide^7^. Although NS has long been a known cause of hypothyroidism in adults and in children, it was associated to cause hypothyroidism in patients with long-standing proteinuria, patients with heavy proteinuria, and patients with steroid-resistant nephrotic syndrome (SRNS). In 2021, Kwong *et al.* 8 concluded in a large retrospective cohort study between 1979 and 2015, that the risk of hypothyroidism was directly related to the severity of proteinuria. Moreover, a study from 2019 which evaluated 317 NS patients stated that patients with thyroid dysfunction expressed a higher levels of proteinuria compared to NS patients with normal thyroid function^9^. In 2014, a study from a teaching hospital in New Delhi, India, which evaluated 20 children with SRNS and 20 healthy controls done by Kapoor *et al.* 10 concluded that a large portion of SRNS children had subclinical nonautoimmune hypothyroidism. It has been known that the ultimate treatment of nonautoimmune hypothyroidism due to NS with no other cause is bilateral nephrectomy, which will stop the loss of thyroid hormones in the urine and thus normalize the thyroid function test^11^.

This presented the case of an unusual early-stage combination of hypothyroidism and NS with no background of previous NS or proteinuria and no previous history of thyroid disorder is the first of its kind reported from Somalia. Our patient’s lower limb edema, along with proteinuria and low level of albumin were suggestive of NS. His thyroid function tests along with generalized muscle ache on admission were also suggestive of overt hypothyroidism due to NS. The patient was hospitalized on the background of NS and hypothyroidism, and after administering ramipril, methylprednisolone, furosemide, aspirin, and atorvastatin and thyroid replacement therapy with close monitoring for 3 weeks, the patient’s clinical condition improved drastically and he was discharged from the hospital.

In 2008, Chandurkar *et al.* 12 reported a case of a 44-year-old woman with long-standing hypothyroidism and heavy proteinuria. They described that the patient lost a significant amount of T4 in the urine which subsequently caused to increase her levothyroxine treatment dose. In contrast, our patient had no history of long-standing hypothyroidism and his proteinuria was less compared to their patient. In 2015, Benvenga *et al.* 13 described 2 case reports in which previously known hypothyroidism patients had required higher doses of levothyroxine treatment to maintain a euthyroid state. They concluded that digestive diseases alone should not be focused in case of increased requirement levothyroxine doses in hypothyroidism patients who previously maintain a euthyroid state and NS should also be suspected and included in the workup.

In 2021, Benido Silva and colleagues described a 23-year-old male patient with severe NS and subsequent complications with overt hypothyroidism which required thyroid hormone replacement. They stated that after the bilateral nephrectomy the patient’s thyroid function tests improved and levothyroxine medication was discontinued^5^.

## Conclusions

Hypothyroidism in NS is a rare entity but it can be encountered in clinical practices. However, hypothyroidism in the early stages of NS is a very rare entity and physicians should be aware that hypothyroidism can be seen at any stage of NS.

## Ethical approval and consent for publication

Mogadishu Somali Turkish Training and Research Hospital Ethics Committee waived approval for this case report.

## Consent

Written informed consent was obtained from the patient for publication of this case report. A copy of the written consent is available for review by the Editor-in-Chief of this journal on request.

## Sources of funding

The authors declare no funding source.

## Author contribution

All authors contributed to this manuscript whether the conception, its drafting, and revising the final manuscript.

## Conflicts of interest disclosure

None.

## Guarantor

Mohamed Osman Omar Jeele.

## Data availability statement

The data is available from the corresponding author and can be accessed if requested.

## Provenance and peer review

Not commissioned externally peer-reviewed.
